# Therapeutic Effects of Bee Venom on Immunological and Neurological Diseases

**DOI:** 10.3390/toxins7072413

**Published:** 2015-06-29

**Authors:** Deok-Sang Hwang, Sun Kwang Kim, Hyunsu Bae

**Affiliations:** 1 Department of Korean Medicine Obstetrics and Gynecology, College of Korean Medicine, Kyung Hee University, 26 Kyungheedae-ro, Dongdaemun-gu, Seoul 130-701, Korea; E-Mail: soulhus@gmail.com; 2 Department of Physiology, College of Korean Medicine, Kyung Hee University, 26 Kyungheedae-ro, Dongdaemun-gu, Seoul 130-701, Korea; E-Mail: skkim77@khu.ac.kr

**Keywords:** Bee Venom, immunological diseases, neurological diseases

## Abstract

Bee Venom (BV) has long been used in Korea to relieve pain symptoms and to treat inflammatory diseases, such as rheumatoid arthritis. The underlying mechanisms of the anti-inflammatory and analgesic actions of BV have been proved to some extent. Additionally, recent clinical and experimental studies have demonstrated that BV and BV-derived active components are applicable to a wide range of immunological and neurodegenerative diseases, including autoimmune diseases and Parkinson’s disease. These effects of BV are known to be mediated by modulating immune cells in the periphery, and glial cells and neurons in the central nervous system. This review will introduce the scientific evidence of the therapeutic effects of BV and its components on several immunological and neurological diseases, and describe their detailed mechanisms involved in regulating various immune responses and pathological changes in glia and neurons.

## 1. Introduction

Bee Venom (BV) therapy is a form of medicine originated from the ancient Greece and China. Several scientific reports suggesting the anti-rheumatic and anti-inflammatory effects of BV have been published for a hundred years [1,2]. In Korea, BV has long been used to relieve pain and to treat various diseases, such as arthritis, rheumatism, herniation nucleus pulpous, cancer, asthma, and skin diseases [3,4,5]. It is administered systemically or in the form of chemical stimulation of acupoints, so called “BV acupuncture” or “apipuncture”. BV is known to contain many active components, including peptides (e.g., melittin and apamin), enzymes (e.g., phospholipase A_2_ (PLA_2_)), and small molecules (e.g., histamine). Recent studies suggested further that BV and BV-derived active components might have potent therapeutic effects on refractory immunological and neurodegenerative diseases including allergic disorders, autoimmune diseases, amyotrophic lateral sclerosis (ALS), and Parkinson’s disease (PD) [3,6,7,8,9], however well-controlled, randomized clinical studies are still insufficient.

In this review, the underlying mechanisms of BV-induced regulation of immune responses as well as of neuronal and glial pathology in refractory immunological and neurological diseases will be discussed, based mainly on the articles that have been published in the last decade. In addition, the therapeutic effects and mechanisms of BV-derived active components, especially focusing on PLA_2_, melittin and apamin will be introduced. Finally, we will comment on the future perspectives in the research area of BV therapy.

## 2. Therapeutic Effects of Bee Venom on Immunological Diseases

### 2.1. Effects on Allergic Disorders

The initial event responsible for the development of allergic disorders, such as asthma, allergic rhinoconjunctivitis, and atopic eczema, is the generation of allergen-specific CD4^+^ T cells [10]. In a general view, allergy is a T helper 2 (Th2) cells-mediated disease that involves the hyperproduction of specific immunoglobulin E (IgE) antibodies to which interleukin-4 (IL-4) and IL-13, the key Th2-specific cytokines, mainly contribute [11].

BV therapy is a kind of allergen-specific immunotherapy (SIT) that has been carried out for a long time. Although the mechanism of SIT remains poorly understood, hitherto several features, including modifications of antigen presenting cells (APCs), T cells, and B cells, as well as both the number and the function of effector cells that mediate the allergic response have been clarified [12]. In clinical trials, it was reported that SIT increases the production of IL-10 by APCs, including B cells, monocytes, and macrophages [12]. The efficacy of SIT has been emphasized in insect venom allergy and respiratory allergies. BV immunotherapy has early and late influences on major cells of allergic inflammation [10]. Venom immunotherapy induces a monocyte activation characterized by a delayed overproduction of IL-12 and tumor necrosis factor alpha (TNF-α), which are cytokines related to the inhibition of Th2 cells [13]. BV immunotherapy is known to generate IL-10 and transforming growth factor beta (TGF-β), which potently suppresses IgE production and increases IgG4 and IgA, simultaneously [14]. Our previous study demonstrated that BV induces Th1 lineage development from CD4^+^ T cells without affecting Th2 cells, by increasing the expression of a Th1-specific cytokine, interferon gamma (INF-γ), via an upregulation of Th1-specific transcription factor, T-bet [15].

CD4^+^CD25^+^Foxp3^+^ regulatory T cells (Tregs) play a pivotal role in the maintenance of tolerance in the immune system and are involved in the control of transplantation tolerance, tumor immunity, allergy, and infection [16,17]. Tregs could regulate allergic disorders through several inhibitory pathways, including suppression of Th2 immune responses, of Th17 cells, and of T cell migration to tissues [11]. It has been suggested that an essential step in successful BV immunotherapy is associated with the presence of Tregs, which secrete IL-10, consequently inhibiting the secretion of cytokines IL-4, IL-5, and IL-13 from Th2 cells, in turn impeding specific IgE production [18]. For example, our recent study showed that BV treatment increased Treg populations, augmented the production of IL-10, and suppressed the production of Th1, Th2, and Th17-related cytokines, resulting in bronchial inflammation with a reduction in the degranulation of mast cells and eosinophils in an OVA-induced allergic asthma murine model [3]. Taken together, the immunological mechanisms of BV immunotherapy include a shift toward Th1 cytokines, an increase in the number of peripheral Tregs, and an upregulation of different markers expressed on CD4^+^ T cells [10,19].

The most important allergen in BV is PLA_2_, which induces rapid leukotriene C4 production from purified human basophils within 5 min, while IL-4 expression and production is induced at later time-points without histamine release [20]. Direct injection of the BV-derived PLA_2_ (bvPLA_2_) into inguinal lymph nodes enhanced allergen-specific IgG and T-cell responses and stimulated the production of the Th1-dependent subclass IgG2a [21]. Melittin, a major peptide component of BV, is reported to trigger lysis of a mast cells, which can lead to the release of histamine and other intracellular components into surrounding tissues [18]. In our unpublished data [22], bvPLA_2_ increased Treg population *in vitro* and *in vivo* more potently than BV, resulting in prevention of ovalbumin-induced allergic asthma in mice with suppression of various effector cells, such as eosinophils, lymphocytes, and macrophages, and of Th2 cytokines and serum IgE.

### 2.2. Effects on Autoimmune and Inflammatory Diseases

Autoimmune diseases, such as rheumatoid arthritis, systemic lupus erythematosus, and multiple sclerosis, have been understood to be Th1-dominant diseases, however, the important roles of Th17 cells and Tregs in autoimmune diseases have recently emerged [23]. Rheumatoid arthritis is a common autoimmune disease, yet current conventional therapies are not always successful [24]. BV has been traditionally used to treat chronic inflammatory diseases, including rheumatoid arthritis [4]. Especially, the anti-rheumatic and anti-inflammatory effects of BV have been understood as of one hundred years ago [1,2]. Previous study has demonstrated that BV injection into the Zusanli acupoint has both anti-inflammatory and anti-nociceptive effects on Freund’s adjuvant-induced arthritis in rats [25]. Effect of combined application of bee-venom therapy and medication is superior to the simple use of medication in relieving rheumatoid arthritis and might reduce the commonly-taken doses of Western medicines [26]. These anti-arthritis effects have been reported in several arthritis models, and these effects of BV might be associated with melittin, a major peptide component of BV, which has anti-inflammatory and anti-arthritis properties, and inhibitory activity on nuclear factor kappaB (NF-κB) [5].

We previously examined the effects of BV on the nitric oxide (NO) generation by lipopolysaccharide (LPS) or sodium nitroprusside (SNP) in RAW264.7 macrophages, and the expression of inducible nitric oxide synthase (iNOS), cyclooxygenase 2 (COX-2), NF-κB and mitogen-activated protein kinase (MAPK) with RT-PCR in LPS stimulated RAW 264.7 cells. The results showed that BV suppressed NO production and decreased the level of iNOS and COX-2 expression, possibly through suppressing NF-κB and MAPK [27]. We also performed microarray analysis to evaluate the global gene expression profiles of macrophage cell treated with BV. We found that BV decreased the expression of various genes, including mitogen-activated protein kinase kinase kinase 8 (MAP3K8), TNF, suppressor of cytokine signaling 3 (SOCS3), TNF-receptor-associated factor 1 (TRAF1), JUN, and CREB binding protein (CBP), related to the inflammatory effects, which occur in LPS-treated RAW264.7 cells [28]. Other studies support these observations. For example, BV and melittin prevent LPS- or SNP-induced NO and prostaglandin E2 production via c-Jun N-terminal kinase (JNK) pathway dependent inhibition of NF-κB [29]. BV also suppressed adjuvant-induced arthritis in rats by targeting TNF-α and NF-κB activation [30]. These findings indicate that BV may have anti-inflammatory effects in rheumatoid arthritis.

Lupus nephritis, a serious complication of systemic lupus erythematosus, is mediated by the glomerular inflammation involving the production of autoantibodies against the nucleus and of cytokines/chemokines, which ultimately results in irreversible renal damage [31,32]. New Zealand Black/White F1 female mice age-dependently develop autoimmune disease, which is characterized by glomerulonephritis, proteinuria, and renal dysfunction [33]. Using this animal model, we showed that BV treatment significantly delayed the development of proteinuria, prevented renal inflammation, reduced tubal damage, and decreased immune deposits in the glomeruli, and these results are closely associated with a BV-induced increase in splenic Tregs and decrease in renal proinflammatory cytokines, TNF-α and IL-6 [8]. These results suggest that BV therapy has the potential to modulate autoimmune response in lupus nephritis, possibly by enhancing Tregs and suppressing renal inflammation.

Multiple sclerosis is a chronic inflammatory disease of the central nervous system (CNS) that affects more than one million people worldwide. Its clinical symptoms include ataxia, loss of coordination, sensory impairment, cognitive dysfunction, and fatigue [34]. The pathogenesis of multiple sclerosis is known to be an autoimmune T cell responses, in which Th1 and Th17 cells are critically involved [35]. An animal model of experimental autoimmune encephalomyelitis (EAE) has been widely used for the study of multiple sclerosis, because clinical and pathological features of EAE are very similar to those of multiple sclerosis. We previously demonstrated that BV treatment has a neuroprotective effect against immune cell infiltration and Th1/Th17 differentiation via increasing Tregs in EAE mouse model [36]. Very recently, another research group has also reported that BV acupuncture attenuates the development and progression of EAE in rats by upregulating Tregs and suppressing Th1/Th17 cell responses [37]. These results suggest that BV has the potential to become a therapeutic agent for multiple sclerosis, and warrants further investigation of BV and bvPLA_2_ as potent modulators of autoimmune T cell responses in the CNS.

## 3. Therapeutic Effects of Bee Venom on Neurological Diseases

Parkinson’s disease (PD) is one of the most common progressive neurodegenerative disorders, which is characterized clinically by bradykinesia, resting tremor, rigidity, and disturbances in posture and gait resulting from the selective, irreversible loss of dopaminergic (DA) neurons in the substantia nigra (SN) and their terminals in the striatum [38,39]. Activated microglia, innate immune cells in the CNS, near the degenerating DA neurons is known to be a key mediator of neuroinflammation in PD [39,40]. BV acupuncture was reported to be anti-inflammatory and anti-neurodegenerative, and to improve motor symptoms in PD clinical trials [6]. In a 1-methyl-4-phenyl-1,2,3,6-tetrahydropyridine (MPTP)-induced mouse model of PD, BV improved the survival percentage of tyrosine hydroxylase^+^ cells to 70% on day 1 and 78% on day 3 compared with normal mice, and reduced expression of the inflammation markers macrophage antigen complex-1 (MAC-1) and iNOS in the SN [41]. Our previous study also revealed that modulation of peripheral immune tolerance by Tregs may contribute to the neuroprotective effect of BV in the MPTP animal model of PD [42]. Recently, apamin, a specific component of BV, was also shown to have a protective effect in animal models of PD [43].

In an animal model of amyotrophic lateral sclerosis (ALS), mutant human superoxide dismutase 1 (hSOD1) transgenic mice, BV acupuncture inhibited microglia activation and phospho-p38 MAPK expression in the CNS, resulting in improvement of motor activity [9]. It also has been reported that melittin ameliorated the inflammation of lung and spleen in an ALS animal model [44]. BV might be helpful in reducing glutamatergic cell toxicity, which has been reported in many neurodegenerative diseases, including PD, Alzheimer’s disease, and ALS, through the inhibition of MAP kinase activation (e.g., JNK, ERK, and p38) following exposure to glutamate [45]. Although scientific evidence is still limited in this research area, we strongly believe that the aforementioned results may lead to future advanced studies, elucidating the therapeutic effects of BV and its active components on various neurodegenerative diseases and its underlying mechanisms. Indeed, in several preliminary results, we found that BV and BV-derived active components ameliorate Alzheimer’s disease, Parkinson’s disease, and chronic neuropathic pain by modulating peripheral immune and inflammatory responses, as well as modulating central glial activation.

## 4. Conclusions and Perspectives

In this review, we introduced the therapeutic effects of BV and its major components on immunological and neurological diseases, and discussed its underlying mechanisms. We propose that BV is a strong immune modulator that may subsequently affect the CNS glia and neurons. BV also seems to play a role in maintaining homeostasis in our body’s immune system and nervous system, because BV therapy can regulate two immunologically opposite conditions, *i.e.*, allergic disorders (Th2 dominant) and autoimmune diseases (Th1 dominant). It remains to be understood how the same treatments of BV or BV-derived active components could modulate both conflicting diseases. Thus, other T cell populations, such as Th17 cells and Tregs, have emerged as a key players in BV-induced modulation of immune and nervous system. Th17 cells are known to play an important role in the pathogenesis of autoimmune, as well as allergic, diseases [46,47]. In contrast, Tregs inhibits activation of both Th1 and Th2 cells, and of Th17 cells, thereby suppressing autoimmune and allergic diseases [11,35]. Indeed, several recent studies reported that BV or bvPLA_2_ could upregulate peripheral Tregs and/or suppress Th17 responses in various animal models of both diseases [3,8,36,37,48]. Further studies on this issue might shed light on our understandings of such homeostatic therapeutic effects of BV.

In addition, it should be noted that BV is called a “double-edged sword” having nociceptive and anti-nociceptive effects [49], and BV itself could act as a strong allergen. BV induces the release of either of histamine or leukotriene C4 in skin of beekeepers [50], and bvPLA_2_, the major allergen of BV components [51,52], induces a PLA_2_-specific IgE immune responses in mice [53], although BV- and bvPLA_2_-induced Th2 cell immunity and specific IgE production might be protective [54,55]. We also observed that a high dose of BV (2.5 mg/kg, s.c.) treatment could exacerbate oxaliplatin-induced neuropathic pain in rats, whereas low doses of BV (0.25 and 1.0 mg/kg, s.c.) strongly alleviate pain [56]. Thus, the optimal dose and treatment method without side effects should be determined in each disease conditions. Future studies including experimental elucidation of detailed cellular/molecular mechanisms, and well-controlled, randomized clinical trials will lead to a potential therapeutic alternative for treating refractory immunological and neurological diseases.
